# 

**Published:** 1856-12

**Authors:** 


### CONTENTS.

**ORIGINAL COMMUNICATIONS.**

| | Page. |
|---|---|
| Surgical Cases, | 400 |
| Case of Parturition, | 403 |
| Case of Labor | 405 |
| Case of Epilepsy, | 407 |
| Case of Chronic Mortification, | 409 |
| Phytolacca Decandra, | 414 |

SELECTIONS.

| | Page. |
|---|---|
| Introductory of Prof. Marsh, | 418 |
| Transactions Baltimore Pathological Society, | 426 |
| The Use of Epispastics, | 450 |
| Effects of Dentition, | 455 |
| Surgical, | 461 |
| Congenital Non-Expansion of the Lungs, | 462 |
| Veratrum Viride, | 468 |
| Menorrhagia, | 472 |
| Menstruation in Old Age, | 472 |
| Editorial, | 473 |

